# Combined Metabolomics and Biochemical Analyses of Serum and Milk Revealed Parity-Related Metabolic Differences in Sanhe Dairy Cattle

**DOI:** 10.3390/metabo14040227

**Published:** 2024-04-16

**Authors:** Zixin Liu, Aoyu Jiang, Xiaokang Lv, Dingkun Fan, Qingqing Chen, Yicheng Wu, Chuanshe Zhou, Zhiliang Tan

**Affiliations:** 1 Key Laboratory for Agro-Ecological Processes in Subtropical Region, Institute of Subtropical Agriculture, Chinese Academy of Sciences, Changsha 410125, China; liuzixin20@mails.ucas.ac.cn (Z.L.); jiangaoyu21@mails.ucas.ac.cn (A.J.); lvxk@ahstu.edu.cn (X.L.); 82101221244@caas.cn (D.F.); 2018391005@st.gxu.edu.cn (Q.C.); wuyicheng19@mails.ucas.ac.cn (Y.W.); zltan@isa.ac.cn (Z.T.); 2 University of the Chinese Academy of Sciences, Beijing 100049, China; 3 College of Animal Science, Anhui Science and Technology University, Bengbu 233100, China

**Keywords:** primiparous, multiparous, cows, metabolism, lactation

## Abstract

The production performance of dairy cattle is closely related to their metabolic state. This study aims to provide a comprehensive understanding of the production performance and metabolic features of Sanhe dairy cattle across different parities, with a specific focus on evaluating variations in milk traits and metabolites in both milk and serum. Sanhe dairy cattle from parities 1 to 4 (S1, n = 10; S2, n = 9; S3, n = 10; and S4, n = 10) at mid-lactation were maintained under the same feeding and management conditions. The milk traits, hydrolyzed milk amino acid levels, serum biochemical parameters, and serum free amino acid levels of the Sanhe dairy cattle were determined. Multiparous Sanhe dairy cattle (S2, S3, and S4) had a greater milk protein content, lower milk lactose content, and lower solids-not-fat content than primiparous Sanhe dairy cattle (S1). Moreover, S1 had a higher ratio of essential to total amino acids (EAAs/TAAs) in both the serum and milk. The serum biochemical results showed the lower glucose and total protein levels in S1 cattle were associated with milk quality. Furthermore, ultra-high-resolution high-performance liquid chromatography with tandem MS analysis (UPLC-MS/MS) identified 86 and 105 differential metabolites in the serum and milk, respectively, and these were mainly involved in amino acid, carbohydrate, and lipid metabolism. S1 and S2/S3/S4 had significantly different metabolic patterns in the serum and milk, and more vitamin B-related metabolites were significantly higher identified in S1 than in multiparous cattle. Among 36 shared differential metabolites in the serum and milk, 10 and 7 metabolites were significantly and strongly correlated with differential physiological indices, respectively. The differential metabolites identified were enriched in key metabolic pathways, illustrating the metabolic characteristics of the serum and milk from Sanhe dairy cattle of different parities. L-phenylalanine, dehydroepiandrosterone, and linoleic acid in the milk and N-acetylornithine in the serum could be used as potential marker metabolites to distinguish between Sanhe dairy cattle with parities of 1–4. In addition, a metabolic map of the serum and milk from the three aspects of carbohydrates, amino acids, and lipids was created for the further analysis and exploration of their relationships. These results reveal significant variations in milk traits and metabolites across different parities of Sanhe dairy cattle, highlighting the influence of parity on the metabolic profiles and production performance. Tailored nutritional strategies based on parity-specific metabolic profiles are recommended to optimize milk production and quality in Sanhe cattle.

## 1. Introduction

The production performance of dairy cattle is affected by their metabolic state and is closely related to parity [1]. Primiparous cattle are affected by stressful events, such as parturition, lactation, and milking for the first time. Therefore, they differ significantly from multiparous cattle, which have adapted to the calving and lactating processes in terms of external behavior, body metabolism, and milk traits [2,3,4]. In particular, differences in milk quality according to parity have been observed in various cow breeds. Due to distinct metabolic requirements between primiparous and multiparous cows, the milk yield of primiparous Holstein cows is significantly lower than that of multiparous Holstein cows, which have undergone multiple calving experiences [5,6]. While the fat content remains unaffected, there is a small but significant decrease in the protein content associated with an increase in parity [7]. Similarly, the milk yield of Brown Swiss cattle also shows a trend of increasing with increased parity, and the milk protein levels peak in the second parity [8]. However, the milk fat levels do not change with parity, as the amount of milk produced and feed consumed increases with the advancing age of the cow, particularly from the first to subsequent lactations [8]. As the first dairy/meat dual-purpose breed independently cultivated in China, Sanhe cattle were crossbred from several exotic cattle breeds, including Simmental, Siberian, and Inner Mongolian cattle. Sanhe cattle are widely farmed in high-latitude areas, such as the Inner Mongolia Province of China, because of their strong adaptability, rough feed tolerance, and cold tolerance [9]. Compared with globally distributed dairy cattle breeds, such as Holstein, most studies of Sanhe, a new breed unique to China, have focused on genetic performance [9,10,11]. However, there is a lack of comprehensive research on the production performance patterns across the entire Sanhe cattle herd. Furthermore, existing studies on the correlation between production performance and parity in both purebred and crossbred dairy cattle are limited in depth. Most of these studies have focused on observed and measured apparent production indicators, failing to delve into the underlying metabolic reasons for variations in production performance across different parities. In the face of the current situation in which Sanhe dairy cattle are now being gradually promoted for breeding, it is necessary to conduct more in-depth observation and research on the production performance of Sanhe dairy cattle. We hypothesized that Sanhe cattle also had a performance pattern with parity variation like other high-yielding dairy cows. Therefore, it is necessary to clarify the milk production rules among different parity in the Sanhe dairy herd and to investigate which metabolic changes are behind the differences in the performance of cows with different parity, which are necessary for the precise feeding management and efficient breeding of Sanhe cattle in the future.

Metabolic status and milk composition are subject to profound changes during lactation [12]. Metabolites in the blood are closely related to milk synthesis because the precursors of milk components in the blood are the material basis for converting dietary nutrients into milk components in dairy cattle [13]. Glucose, amino acids, fatty acids, and other milk precursors in the blood circulating through the mammary glands are absorbed by mammary epithelial cells via specific transporters, such as amino acid transporters and glucose transporters, to further synthesize lactose, milk protein, milk fat, and other nutrients [14,15]. Although the link between blood and milk has been relatively well studied for specific individual nutrients, such as acetate, β-hydroxybutyric acid, and triacylglycerol [16,17,18], there is a lack of research on the metabolic link between the two bodily fluids and a lack of an overall understanding of most other substances.

In this study, our objective is to systematically investigate the production performance and metabolic characteristics of Sanhe dairy cattle at different parities and explore the correlation between them. Specifically, we aimed to assess the milk quality of Sanhe dairy cattle with parities ranging from 1 to 4 and to elucidate the associated changes in amino acid profiles in both the milk and serum. These data will provide new insights into the metabolic function and production performance of this new breed of cattle, and these multivariate analysis strategies can be used as a reference for dairy cattle metabolism research.

## 2. Material and Methods

### 2.1. Animals, Housing, and Experimental Design

The Sanhe dairy cattle used in this study were obtained from Hulunbuir Agricultural Reclamation Xiertala Farm and Ranch Co., Ltd. (Hulunbuir City, China). Thirty-nine Sanhe dairy cattle with a parity of 1–4 at similar lactation stages were divided into four groups. The S1 group comprised 10 Sanhe dairy cattle in the first parity with 146 ± 7 (mean ± SD) days in milk (DIM), the S2 group comprised 9 Sanhe dairy cattle in the second parity with 151 ± 6 DIM, the S3 group comprised 10 Sanhe dairy cattle in the third parity with 149 ± 8 DIM, and the S4 group comprised 10 Sanhe dairy cattle in the fourth parity with 147 ± 11 DIM. The cattle were fed a total mixed ration and had ad libitum access to water. The basal dietary ingredients and nutrient compositions are consistent with those used in previously published studies and are shown in Supplementary file 1: Table S1 [19]. The cattle were milked three times daily at 0530, 1330, and 1930 h.

### 2.2. Diet and Feed Ingredients

Feed offered and refused was collected for nutrient analysis and dry matter intake. The feed samples were oven-dried at 65 °C and ground to pass through a 1 mm sieve. The gross energy was determined using an isothermal automatic calorimeter (5E-AC8018, Kaiyuan Instruments Co., Ltd., Changsha, China), and the dry matter, crude protein, ether extract, ash, calcium, and phosphorus were determined according to Association of Official Analytical Chemists methods (AOAC, 2002). The neutral detergent fiber and acid detergent fiber content were determined using an automatic fiber analyzer (FT12, Gerhardt, Bonn, Germany) according to a previously published method [20].

### 2.3. Blood Variables

Blood samples were collected from the tail vein using 5 mL vacutainer tubes (GA10001, Sanli, Changsha, China), with two tubes collected from each cow 1 h before feeding. After standing for 2 h, the blood collection tube was centrifuged at 3000 rpm for 10 min at 4 °C. The upper 2 mL of serum was then taken from each tube (3810X, Eppendorf, Germany) and divided into three portions, which were immediately frozen in liquid nitrogen and stored at −80 °C for subsequent analysis of serum biochemical parameters, free amino acids, and metabolomics for the S1 (S1-B), S2 (S2-B), S3 (S3-B), and S4 (S4-B) groups. Blood biochemical parameters, including glucose (04404483190, Roche, Switzerland), albumin (05599261190, Roche, Switzerland), total protein (03183734190, Roche, Switzerland), blood urea nitrogen (04460715190, Roche, Switzerland), triglyceride (11877771216, Roche, Switzerland), cholesterol (03039773190, Roche, Switzerland), high-density lipoprotein cholesterol (04399803190, Roche, Switzerland), low-density lipoprotein cholesterol (03038866322, Roche, Switzerland), lipase (03029590322, Roche, Switzerland), and ammonia (20766682322, Roche, Switzerland) were analyzed using an automatic biochemical analyzer (Hitachi 7600; Hitachi, Tokyo, Japan) with commercial kits. Serum free amino acid concentrations were determined using the principle of post-column derivatization. An amount of 1 mL of 8% 5-sulfosalicylic acid (S2130, Sigma-Aldrich, St. Louis, MO, USA) was added to 1 mL of serum sample to precipitate the protein [21]. After mixing and centrifuging at 10,000 rpm for 5 min, 1 mL of the resulting supernatant was collected and passed through a 0.22 μm filter membrane. The free amino acid concentration was determined by an amino acid analyzer (Hitachi L8900).

### 2.4. Milk Production and Composition

At 0530, 1330, and 1930 h, 50 mL of milk was collected and mixed at a ratio of 4:3:3. The mixture was divided into three portions. Potassium dichromate (P112163, Aladdin, China) was added to one portion for milk traits determination, and the other two portions were immediately frozen in liquid nitrogen and stored at −80 °C for hydrolyzed milk amino acid content determination and metabolomics analysis (S1-M, S2-M, S3-M, and S4-M). The quality of the milk samples was determined using a Basic Unit MilkoScan FT +200 Type 76150 instrument (Foss Electric, Hillerod, Denmark), and the somatic cell count was determined using a Fossomatic FC Type 79910 instrument (Foss Electric). The milk samples were mixed with 6 mol/L hydrochloric acid (HCL) in an ampoule bottle at a ratio of 1:1 (*v*:*v*), hydrolyzed at 110 °C for 24 h, and then diluted to 50 mL. An amount of 1 mL of the solution was dried to remove the solvent, redissolved with 1 mL of 0.01 N HCL, and passed through a 0.22 μm filter membrane. The hydrolyzed milk amino acid content was then determined using an amino acid analyzer (Hitachi L8900).

### 2.5. Metabolomics Analysis by UPLC-MS/MS

Untargeted metabolomic analysis of serum and milk samples was performed using UPLC-MS/MS. An amount of 100 microliters of internal standard solution was mixed with the samples in 400 μL of methanol (−20 °C). After centrifuging at 4 °C for 10 min at 12,000 rpm, 500 μL of the supernatant was collected and concentrated to dryness under a vacuum. The samples were then redissolved with 150 μL of 80% methanol and centrifuged at 4 °C for 10 min at 12,000 rpm to obtain the supernatant used for UPLC-MS/MS analysis. A liquid chromatographic separation assay was performed using an ACQUITY UPLC^®^ HSS T3 column (2.1 × 150 mm × 1.8 μm; Waters Co., Ltd., Milford, MA, USA). The autosampler temperature was set to 8 °C, 2 μL was injected at a flow rate of 0.25 mL/min, and gradient elution was performed at a column temperature of 40 °C. The mobile phase was 0.1% formic acid in water or 0.1% formic acid in acetonitrile in positive ion mode and 5 mM ammonium formate in water and acetonitrile in negative ion mode. Metabolite detection was performed using a Thermo Orbitrap Exploris 120 mass spectrometer (Thermo Fisher Scientific, Waltham, MA, USA) with spray voltages of 3.5 kV and −2.5 kV in positive and negative modes, respectively. The capillary temperature was 325 °C. The Orbitrap analyzer scanned a mass-to-charge ratio (*m*/*z*) range of 100–1000 for a full scan at a mass resolution of 60,000. Data-dependent acquisition was performed for MS/MS experiments using high-energy collision dissociation scans, with cracking rates of 30%, 50%, and 150%. Dynamic exclusion was performed to remove unnecessary information from MS/MS spectra.

### 2.6. Metabolomics Data Processing and Analysis

The raw data were converted into mzXML format using ProteoWizard software (v3.0.8789). The XCMS package in R (v3.3.2) was used for peak identification, filtration, and alignment. The main parameters were set to bw = 5, ppm = 15, peakwidth = c (5, 30), mzwid = 0.015, mzdiff = 0.01, and method = “centWave”. The obtained data matrix of *m*/*z*, retention time, and peak area (intensity) in positive and negative ion modes were used for subsequent analyses. The resulting data matrices were subjected to mean centering and scaled to unit variance. Multivariate principal component analysis (PCA) and partial least squares discriminant analysis (PLS-DA) were performed using SIMCA-P software (version 15.0; Umetrics AB, Umeå, Sweden) and the R package, RoPLS. The total explained variance (R2) and predictability (Q2) were calculated to determine the model validity. The screening conditions for differential metabolites were a molecular weight error < 15 ppm, variable importance in projection > 1, fold_change (FC) value ≥ 1.5 or ≤ 0.667, and *p*-value ≤ 0.05. The *p*-value was obtained using a *t*-test between the two groups for pairwise comparisons and a one-way ANOVA test to screen for differential metabolites between the four parities. Accurate information of the metabolites was then acquired by further matching and annotating them in the Metlin (http://metlin.scripps.edu (accessed on 24 July 2022)) and MoNA (https://mona.fiehnlab.ucdavis.edu/ (accessed on 24 July 2022)) databases, and the database built by BioNovoGene Co., Ltd. (Suzhou, China). Hierarchical clustering analysis was performed using the agglomerate hierarchical clustering method, scaled normalization of the dataset was performed using the pheatmap package in R (v3.6.3), and advanced heatmap plots and receiver operating characteristic curves (ROC) were performed using the OmicStudio tools at https://www.omicstudio.cn (accessed on 15 November 2022). Differential metabolites were identified and classified based on the Human Metabolome Database (HMDB; http://www.hmdb.ca (accessed on 15 September 2022)) and Kyoto Encyclopedia of Genes and Genomes (KEGG) pathway database (http://www.genome.jp/kegg/ (accessed on 17 September 2022)). Metaboanalyst 5.0 (http://www.metaboanalyst.ca/ (accessed on 12 October 2022)), R version 4.0.2, and iPATH 3 (https://pathways.embl.de/ (accessed on 15 October 2022)) were used for metabolic pathway and metabolite network interaction analyses. The source of the metabolites, their relative levels in cows of different parities, the association between the categories and individual metabolites, their position in the metabolic pathway, and the conversion relationships with upstream and downstream metabolites were analyzed, respectively. Correlation networks consisting of differential metabolites with differential indicators detected in serum as well as milk were tested analytically using Mantel’s test, Pearson’s correlation, Spearman’s correlation, and visualized using the R (version 4.3.1) packages linkET and ggplot2. The specific parameters are in the supplementary analysis code section.

### 2.7. Statistical Analysis of Data

All the data were processed using Excel 2019 (Microsoft Corporation, Redmond, WA, USA). Data on feed ingredients, milk quality, serum biochemical parameters, serum free amino acid levels, and hydrolyzed milk amino acid levels of the four groups were analyzed using the Shapiro–Wilk test for normally distributed data, followed by a one-way ANOVA to compare means using the Statistical Package for the Social Sciences software (version 22.0; IBM, Armonk, NY, USA). The Bonferroni adjustment was used to correct for multiple comparisons between categorical variables. Statistical significance was defined as *p* ≤ 0.05, and tendencies as 0.05 < *p* < 0.10.

## 3. Results

### 3.1. Lactation Performance and Milk Traits

The lactation performance and milk traits results for Sanhe dairy cattle of parities 1–4 are shown in Table 1. In the absence of significant differences in body weight and DMI (*p* > 0.1), the milk protein content was significantly higher in groups S2 and S3 than groups S1 and S4 (*p* = 0.001). The lactose content (*p* = 0.009) and solids-not-fat content (*p* = 0.011) were significantly higher in groups S1 than group S1–S4, whereas the four groups showed no significant differences in other lactation performance indicators, including milk yield, somatic cell count, milk fat content, urea nitrogen content, and total solids (*p* > 0.1). The hydrolyzed amino acid analysis results in the milk from Sanhe dairy cattle of different parities are also shown in Table 1. Methionine (*p* = 0.009), tyrosine (*p* = 0.003), and proline (*p* = 0.001) had the highest content in the first parity. At the same time, the total amino acid (TAA), essential amino acids (EAAs), and non-essential amino acids (NEAAs) showed trends toward differences among the groups, with both TAA (*p* = 0.085) and EAA (*p* = 0.076) trending higher in groups S2 and S3 than in group S1, whereas NEAA (*p* = 0.077) had the highest levels in group S1. Moreover, the EAA/TAA ratio significantly differed between groups S2, S3, and S4 and S1 (*p* = 0.019), with group S1 having the lowest EAA/TAA ratio (*p* = 0.003). In addition, the phenylalanine (*p* = 0.087), lysine (*p* = 0.093), and glycine (*p* = 0.088) levels also showed a trend toward being higher in groups S2, S3, and S4 than in group S1. The remaining amino acids were not significantly different among the 1–4 parities (*p* > 0.10).

### 3.2. Serum Biochemical Parameters and Free Amino Acid Levels

The serum biochemical parameters and free amino acid levels of Sanhe dairy cattle of parities 1–4 in are shown in Table 2. Both the glucose (*p* = 0.017) and total protein (*p* = 0.017) levels were lowest in group S1 and group S4. However, the serum ammonia content was significantly different between the groups, with the highest content in group S1 (*p* = 0.002). The triglyceride levels were significantly lower in group S3 than group S4 (*p* = 0.010). In addition, the high-density lipoprotein levels (*p* = 0.047) were significantly higher in groups S1 and S2 than group S4. Other blood biochemical parameters, such as albumin, blood urea nitrogen, cholesterol, low-density lipoprotein, and lipase levels, showed no significant changes among the four parities of Sanhe dairy cattle (*p* > 0.05). For the free amino acid results in the serum, among the amino acids with significant differences between groups, proline showed the lowest levels in group S1 (*p* = 0.014), and methionine (*p* = 0.024), aspartate (*p* = 0.034), alanine (*p* = 0.034), and glycine (*p* = 0.006) all had the highest levels in group S1. In addition, the NEAA and cysteine levels and the EAA/TAA ratio showed trends toward differences between the groups, with NEAA (*p* = 0.053) showing the highest levels in group S3, and the cysteine levels (*p* = 0.081) and the ratio of EAA/TAA (*p* = 0.057) showing the highest values in group S2. The serum levels of the remaining free amino acids showed no significant differences between groups (*p* > 0.10).

### 3.3. Metabolomics Multivariate Analysis of Serum and Milk

**Figure 1:**
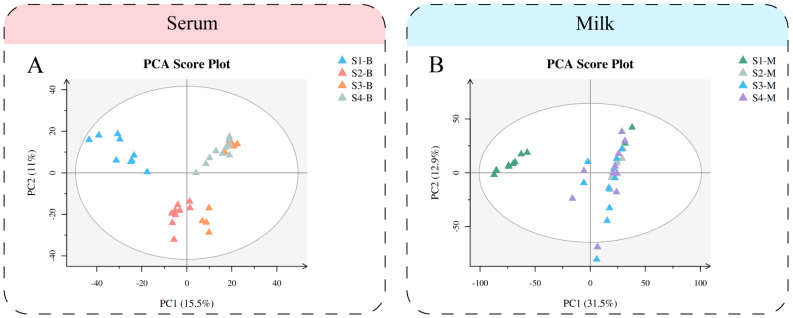
Multivariate statistical analysis of metabolites identified from Sanhe dairy cattle with 1–4 parities. (**A**) Principal component analysis (PCA) score plot of serum samples from S1 to S4 based on untargeted metabolomics. (**B**) PCA score plot of milk samples from S1 to S4 based on untargeted metabolomics. The abscissa PC1 = first principal component and the ordinate PC2 = second principal component.

**Figure 2:**
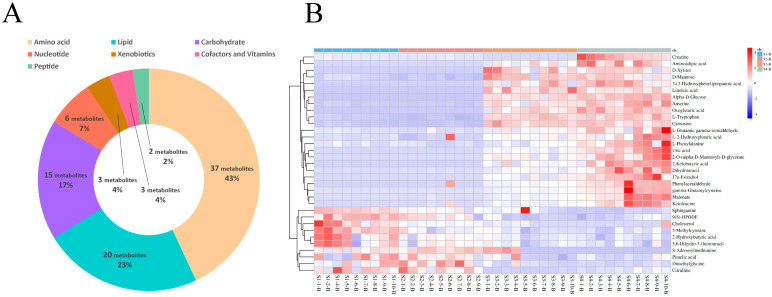
Identification and classification of differential serum metabolites. (**A**) Classification of metabolites from serum based on Kyoto Encyclopedia of Genes and Genomes (KEGG) pathway analysis. (**B**) Hierarchical cluster analysis (HCA) of differential metabolites identified from S1-B, S2-B, S3-B, and S4-B groups.

We performed multivariate statistical analysis and visualized the general clustering pattern and demonstrated the differences between the serum and milk of Sanhe dairy cattle of parities 1–4 (Figure 1). The PCA score plots (Figure 1A) of the serum samples from Sanhe dairy cattle revealed distribution between the four parities. Meanwhile, the PLS-DA model showed that the four groups of serum samples were able to be significantly separated (Figure S1A), while R2 = 0.86 and Q2 = −0.32 showed a good fit (Figure S1C). Similarly, PCA (Figure 2B) and PLS-DA (Figure S1B) were performed on the milk samples from S1 to S4, and the results were all shown to indicate that S1-M had a high degree of separation from the other three groups of milk samples. The values R2 = 0.74 and Q2 = −0.33 from the PLS-DA model for the milk samples show no overfitting (Figure S1D). The verification parameters of the multivariate statistical analysis model are listed in Supplementary file 1: Table S2.

### 3.4. Differential Metabolite Identification in Serum and Milk

For the serum samples from Sanhe dairy cattle of parities 1–4, 86 metabolites were identified using untargeted UPLC-MS/MS (Supplementary file 2: Table S1). Based on their corresponding KEGG superpathways, the identified metabolites were divided into seven categories: amino acids (43%, 37 metabolites), lipids (23%, 20 metabolites), carbohydrates (17%, 15 metabolites), nucleotides (7%, 6 metabolites), xenobiotics (4%, 3 metabolites), cofactors and vitamins (4%, 3 metabolites), and peptides (2%, 2 metabolites; Figure 2A). To further examine the enrichment of the identified metabolites in the serum samples from the four groups, hierarchical cluster analysis (HCA) was performed. The heat map results showed that groups S1-B and S2-B had similar metabolic patterns and groups S3-B and S4-B had similar metabolic patterns (Figure 2B). Among the 33 differential metabolites identified in the serum samples from the four groups, metabolites related to lipid metabolism, such as sphinganine, 9(S)- hydroperoxyoctadecadienoic acid (HPODE), cholesterol, and pimelic acid, were enriched in groups S1-B and S2-B, whereas aromatic amino acids, such as L-tryptophan and L-phenylalanine, were significantly enriched in groups S3-B and S4-B. Furthermore, a pairwise comparison of the serum samples from group S1-B with those from the other groups was performed using HCA heat maps, and 27, 39, and 40 differential metabolites were identified from the S1B/S2-B, S1B/S3-B, and S1-B/S4-B pairs, respectively (Figure S2A,C,E). The differential metabolite details of specific pairwise comparisons from the serum are listed (Supplementary file 2: Tables S3–S5). In addition, the identified differential metabolites for each pair were divided into seven categories based on KEGG pathways, and the resulting fold-change value was visualized to determine the specific change values for each differential metabolite in each pair of comparisons (Figure S2B,D,F). Overall, the levels of differential lipid-related metabolites, such as sphinganine, 9(S)-HPODE, cholesterol, and 2-hydroxybutyric acid, and the nucleotide metabolite, 5-methylcytosine, identified in the pairwise comparison, were higher in S1-B than in the other groups, whereas the levels of the lipid-related metabolite, erucic acid, were lower in the S1-B group in each pair of comparisons. Pairwise comparisons of other parities, such as S2-B vs. S3-B and S3-B vs. S4-B, were also performed, and the results are shown in Figure S1. For the S2-B vs. S3-B comparisons, the differential metabolites were highly discriminative between the two groups, indicating a difference in the metabolic patterns between the two groups (Figure S3A). The heat map results of the S3-B vs. S4-B comparison showed a low degree of separation and enrichment of differential metabolites between the two groups, indicating that the two groups had similar metabolic patterns (Figure S3B).

**Figure 3:**
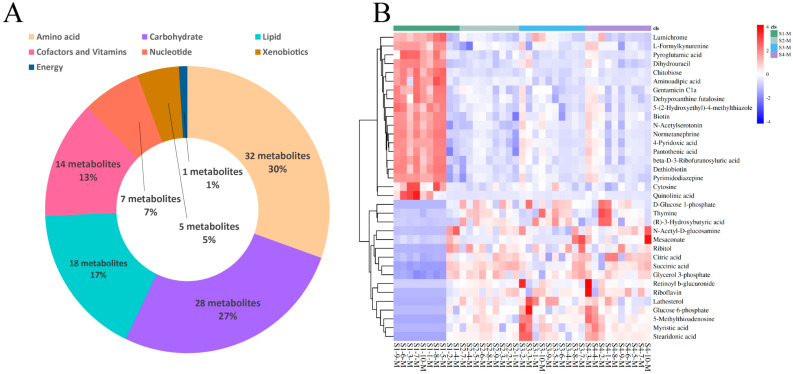
Identification and classification of differential metabolites in milk. (**A**) Classification of metabolites from serum based on Kyoto Encyclopedia of Genes and Genomes (KEGG) pathway analysis. (**B**) Hierarchical cluster analysis (HCA) of differential metabolites identified from the S1-M, S2-M, S3-M, and S4-M groups.

For the four groups of milk samples from Sanhe dairy cattle with parities of 1–4, 105 metabolites were identified using untargeted UPLC-MS/MS (Supplementary file 2: Table S2). Based on the KEGG pathway analysis, these metabolites were divided into seven categories (Figure 3A). Similar to the results for the serum samples, the three most predominant categories were amino acids (30%, 32 metabolites), carbohydrates (27%, 28 metabolites), and lipids (17%, 18 metabolites), followed by cofactors and vitamins (13%, 14 metabolites), nucleotides (7%, 7 metabolites), and xenobiotics (5%, 5 metabolites). In contrast to the serum samples, the least prevalent category for the milk samples was energy (1%, 1 metabolite). HCA was performed on the milk samples from the four groups of cattle, and the results were plotted as a heat map (Figure 3B), which clearly showed 35 differential metabolites in group S1-M compared to the other three groups. Further pairwise comparisons of S1-M with the other three groups and HCA were performed. The heat map results showed that these three pairs were very similar, with 49, 40, and 41 differential metabolites (Supplementary file 2: Tables S6–S8) for the S1-M vs. S2-M, S1-M vs. S3-M, and S1-M vs. S4-M comparisons, respectively (Figure S4A,C,E). The differential metabolites identified between the pairwise comparisons were also classified, and the fold-change values corresponding to the differential metabolites between the groups were calculated (Figure S4B,D,F). The levels of differential metabolites identified in both the lipid and carbohydrate classes (except chitobiose) were lower in the S1-M group than the other groups, whereas those in the cofactors and vitamins class, except riboflavin and retinoyl b-glucuronide, were higher in the S1-M group. Pairwise comparisons of the adjacent milk sample groups were performed using HCA (Figure S3). Few differentially expressed metabolites were identified in the S2-M vs. S3-M (Figure S3C) or S3-M vs. S4-M (Figure S3D) comparisons, with only six and two metabolites identified, respectively. These results suggest that the metabolic activity was similar among the three groups.

### 3.5. Pathway Analysis of Serum and Milk

**Figure 4:**
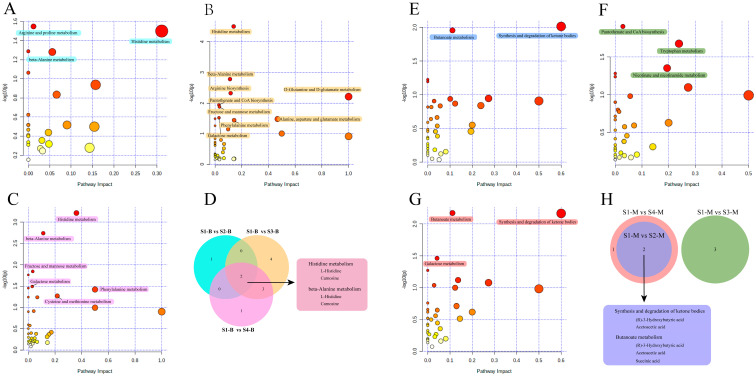
Functional pathway enrichment analysis of differential metabolites in pairwise comparisons of serum and milk samples. (**A**–**C**) Bubble chart analysis of functional pathways from S1-B vs. S2-B, S1-B vs. S3-B, and S1-B vs. S4-B comparisons, respectively. (**E**–**G**) Bubble chart analysis of functional pathways from S1-M vs. S2-M, S1-M vs. S3-M, and S1-M vs. S4-M comparisons, respectively. x-axis, pathway impact; y-axis, −log (*p*). Circles represent metabolic pathways. Darker circles indicate more significant changes in the metabolite levels in the corresponding pathway, whereas the circle size corresponds to the pathway impact score. Significantly enriched functional pathways are marked with colored blocks of the corresponding groups. (**D**,**H**) Venn diagrams of functional pathways shared by groups in pairwise comparisons of serum and milk samples. The colors of the Venn circles correspond to the colors of the significantly enriched pathways of the two-by-two comparison groups. The repetitive functional pathways and their corresponding metabolites are marked with rounded rectangles.

Based on the metabolic pathway information for Bos taurus (cow) as the model organism in the KEGG, functional pathway enrichment analysis was performed for the differential metabolites identified in the serum and milk samples. For pairwise comparisons with samples from the S1 group, functional pathways that were significantly enriched (*p* < 0.05, pathway impact > 0) are marked with different colored blocks in Figure 4. For the serum samples, the differential metabolites identified in other groups compared to S1-B were enriched in multiple metabolic pathways. Among them, the differential metabolites identified in the S1-B vs. S2-B comparison had significant effects on three pathways related to amino acid metabolism: histidine metabolism, beta-alanine metabolism, and arginine and proline metabolism (Figure 4A). The S1-B vs. S3-B comparison had the largest number of enriched pathways among the three groups, and the nine significantly enriched pathways included pantothenate and CoA biosynthesis, D-glutamine and D-glutamate metabolism, and galactose metabolism, which are related to the metabolism of cofactors and vitamins, amino acids, and carbohydrates, respectively (Figure 4B). Meanwhile, the differential metabolites identified from the S1-B vs. S4-B comparison were also significantly enriched in six pathways, including two pathways related to carbohydrate metabolism and four pathways related to amino acid metabolism (Figure 4C). In addition, the pathways enriched in the S1-B vs. S4-B comparison coincided with those of S1-B vs. S3-B, except for the cysteine and methionine metabolism pathways. Statistical analyses and Venn diagrams of the enriched pathways in these three comparisons showed that they were all enriched in histidine metabolism and beta-alanine metabolism pathways. The differential metabolites L-histidine and carnosine were covered by both pathways (Figure 4D). The number of significantly enriched metabolic pathways in the pairwise comparisons of cows with parities of 1–4 were less for the milk samples than the serum samples. The differential metabolites identified in the S1-M vs. S2-M comparison were significantly enriched in butanoate metabolism and the synthesis and degradation of ketone bodies (Figure 4E). The results of the S1-M vs. S3-M comparison showed significant enrichment in three metabolic pathways: pantothenate and CoA biosynthesis, tryptophan metabolism, and nicotinate and nicotinamide metabolism (Figure 4F). For the S1-M vs. S4-M comparison, in addition to the pathways enriched in the S1-M vs. S2-M comparison, the galactose metabolism pathway was also significantly enriched (Figure 4G). A Venn diagram showed that the results of the S1-M vs. S3-M comparison differed from those of the other two comparisons, with no overlapping significantly enriched functional pathways (Figure 4H). The S1-M vs. S2-M and S1-M vs. S4-M comparisons showed overlapping pathways of butanoate metabolism and the synthesis and degradation of ketone bodies, which included the differential metabolites (R)-3-hydroxybutanoate and acetoacetic acid. In addition, succinic acid was identified as a differential metabolite belonging to the butanoate metabolic pathway.

### 3.6. Interaction Analysis of Serum and Milk Metabolites

**Figure 5:**
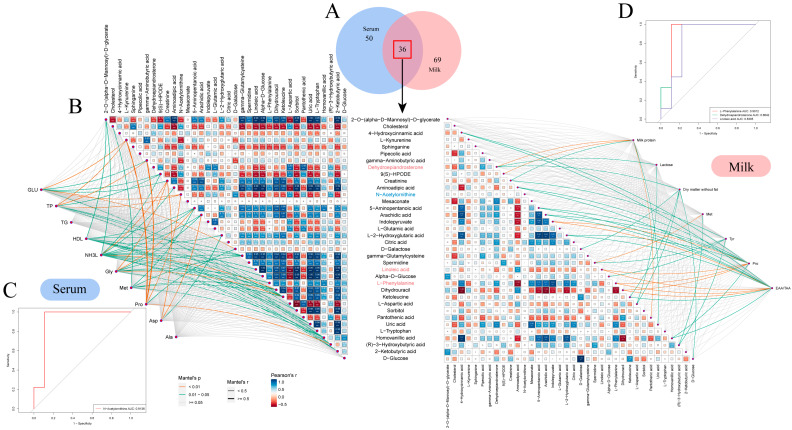
Identification and analysis of common differential metabolites in serum and milk. (**A**) Venn diagrams of identified metabolites in serum and milk samples. Blue and red circles represent milk and serum samples, respectively. (**B**) Correlation network analysis of shared differential metabolites with indicators of difference in serum and milk. On the left, the associations of 36 shared differential metabolites with significant indicators of difference in S1–S4 serum are shown. On the right, the associations of 36 shared differential metabolites with significant indicators of difference in S1–S4 milk are shown. Correlations between metabolites and differential indicators were determined using Mantel’s tests, with the thickness of the connecting line indicating the correlation coefficient, with a thick line indicating a Mantel’s r ≥ 0.5; The color of the connecting line indicates significance, with orange being a highly significant correlation (*p* < 0.01) and green being a significant correlation (0.01 < *p* < 0.05). Pearson’s test tested correlations between shared differential metabolites; box size and color gradient indicate Pearson’s correlation, blue indicates positive correlation, red indicates negative correlation, and white words in the boxes indicate correlation coefficients. Biomarkers in serum are indicated in blue font and biomarkers in milk are indicated in pink font. White text in the boxes represents correlation coefficients, where * denotes 0.01 < *p* < 0.05, ** denotes 0.001 < *p* < 0.01, and *** denotes *p* < 0.001. (**C**) Predictive analyses of metabolites strongly associated with significantly different physiological indicators in serum (AUC > 0.8). (**D**) Predictive analyses of metabolites strongly associated with significantly different physiological indicators in milk (AUC > 0.8).

To study the correlation between the metabolites identified in the serum and milk samples from Sanhe dairy cattle with parities of 1–4, the 86 metabolites identified in the serum samples were compared with the 105 metabolites identified in the milk samples. Thirty-six metabolites were found to be coincident between the two sample types (Figure 5A). In the serum samples, the abundance of metabolites was more similar between the S1-B and S2-B groups, in contrast to the S3-B and S4-B groups, as evidenced by the hierarchical clustering analysis (HCA) results. In the milk samples, the S1-M group was significantly different from groups S2-M, S3-M, and S4-M (Figure S5A). It was also found that metabolites such as D-galactose, D-glucose, ketoleucine, and gamma-glutamylcysteine were all identified at higher levels in serum than in milk. Further functional pathway enrichment analysis of the 36 overlapping metabolites showed that there were seven enriched pathways, including alanine, aspartate, and glutamate metabolism; galactose metabolism; and pantothenate and CoA biosynthesis, which mainly involved three modules, including amino acid metabolism, carbohydrate metabolism, and the metabolism of cofactors and vitamins (Figure S5B). To establish the correlation between the 36 shared differential metabolites and physiological indicators showing significant differences in the serum and milk, we conducted a Mantel’s test analysis and a Pearson’s correlation analysis (Figure 5B). There were 10 metabolites in the serum that strongly correlated with differential physiological indicators, with the exception of D-galactose, which was highly significantly correlated with glucose and significantly correlated with defatted dry matter; most of the metabolites that showed strong correlations with serum indicators of significant differences belonged to the amino acid class, including the following: L-kynurenine was highly significantly correlated with glucose, total protein, and protein (*p* < 0.01); creatinine was significantly correlated with serum ammonia, glycine, and aspartate (*p* < 0.05); N-acetylornithine was highly significantly correlated with glucose, total protein, and aspartate (*p* < 0.01). In addition, serum ammonia was significantly associated with up to 14 metabolites, including a highly significant association with ketoleucine (*p* < 0.01). There were 7 metabolites in the milk that strongly correlated with differential physiological indicators. Among them, 9(S)-HPODE was significantly associated with milk protein (*p* < 0.05), and linoleic acid was significantly associated with proline and EAA/TAA (*p* < 0.05). These metabolites that strongly correlated with differential physiological indicators were further analyzed by ROC analysis to discern whether they could be used as marker metabolites to distinguish between Sanhe dairy cattle with parities of 1–4. Setting an AUC > 0.8 for screening, it was found that the AUC of N-acetylornithine in the serum reached 0.9163 (Figure 5C), while the AUC of L-phenylalanine, dehydroepiandrosterone, and linoleic acid in the milk reached 0.9012, 0.8642, and 0.8395 (Figure 5D), all of which have the potential to be effective marker metabolites for distinguishing between Sanhe dairy cattle with parities of 1–4.

**Figure 6:**
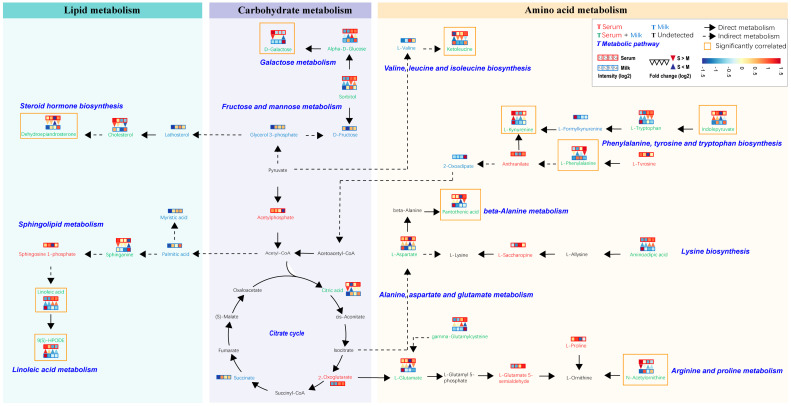
Metabolic networks involving pathways of lipid metabolism, carbohydrate metabolism, and amino acid metabolism consisting of differential metabolites in serum and milk. Metabolites identified in serum and milk samples are marked in red and green, respectively, and metabolites identified in both serum and milk samples are marked in green. In the legend, the four red squares represent serum samples from Sanhe dairy cattle with parities of 1–4, and the four blue squares represent milk samples from Sanhe dairy cattle with parities of 1–4. Blue indicates relatively low levels and red indicates relatively high levels. The color change in the squares is described based on identification intensity (log2) of the metabolite, with blue indicating lower and red indicating higher. The inverted red triangle indicates relatively higher levels of metabolites in serum than in milk, and the blue triangle indicates relatively lower levels of metabolites in milk than in serum. The color change in the triangle is described based on the fold change (log2) of the metabolite between serum and milk, with blue indicating lower and red indicating higher. The lipid metabolism, carbohydrate metabolism, and amino acid metabolism categories are represented by blue, purple, and wheat blocks, respectively. Black solid arrows indicate direct metabolism, and black dashed arrows indicate the presence of more than two intermediate metabolites. The metabolites in the orange boxes are strongly correlated with physiological indicators that are significantly different in serum or milk. S1: first-parity Sanhe dairy cattle; S2: second-parity Sanhe dairy cattle; S3: third-parity Sanhe dairy cattle; S4: fourth-parity Sanhe dairy cattle; S: serum; M: milk.

In order to further explore the metabolic pathways impacted by these shared differential metabolites, we localized their positions in the metabolic network. We combined the results of previous metabolite classifications with the KEGG database to integrate these localized metabolic pathways, which were mainly categorized into three modules, consisting of lipid metabolism, carbohydrate metabolism, and amino acid metabolism (Figure 6). Metabolites found to be highly significantly correlated with differential physiological indicators (marked by orange boxes) were distributed in all three modules. The highest number of metabolites was found in the amino acid metabolism module, mainly affecting the valine, leucine, and isoleucine biosynthesis, phenylalanine, tyrosine, and tryptophan biosynthesis, beta-alanine metabolism arginine and proline metabolism, and galactose metabolism. In addition, the galactose metabolism pathway covers a variety of shared metabolites, including sorbitol, alpha-D-glucose, and D-galactose. We also found that indolepyruvate in the serum was highly significantly negatively correlated with lipid metabolites such as cholesterol, sphinganine, and 9(S)-HPODE in the milk, and significantly positively correlated with amino acid metabolites such as aminoadipic acid, L-phenylalanine, and L-Tryptophan (Figure S6).

## 4. Discussion

### 4.1. Characteristics of Serum and Milk from Sanhe Dairy Cattle with Parities of 1–4

Cow milk is a nutritious natural food, but its quality is affected by many factors, such as the cow breed, parity, lactation stage, season, nutritional level, and feeding management [22,23,24,25,26]. In contrast to the typical pattern observed in many dairy cows where the milk yield increases with parity, this study did not observe statistically significant differences in the milk yield among Sanhe cows of varying parities. The reasons for this phenomenon may be a limited sample size and breed differences. Sanhe cattle, unlike other breeds such as Holsteins, exhibit genetic and physiological differences, which may partially account for the observed lack of change in the milk yield. Additionally, this study focused on mid-lactation Sanhe cows, which may undergo physiological adjustments during this stage to meet the demands of lactation, resulting in a more stable milk yield. These physiological adaptations may blur the differences in the milk yield between cows of different lactation numbers. Increasing the content of milk fat and milk protein, which are important nutritional factors in milk, is the goal of dairy farming in many countries, as these factors are the basis for the price of raw milk [27]. In this study, we report that, compared with primiparous Sanhe dairy cattle (S1), multiparous Sanhe dairy cattle (S2, S3, and S4) had higher levels of milk protein, lower levels of lactose, and lower solid nonfat. In particular, changes in milk protein followed the same trend as observed in previous studies of Holstein cattle [28]. This may be because the growth of the cows was almost complete, and the milk production potential was significantly increased by the second lactation period [29]. At the beginning of the second and subsequent lactation periods, the metabolic reserves signal the productive/reproductive axis to induce differential nutrient partitioning in primiparous and multiparous cows, resulting in metabolic differences [30,31]. The physical functioning of the entire herd or a specific group of cattle can be assessed by detecting and analyzing the metabolic status of individual cattle at specific stages. This is beneficial for making further decisions about production management.

Serum contains the major nutrients carried by the blood, and the contents of the serum can reflect the nutritional metabolism [32], digestive metabolism [33], immune metabolism [34], and other metabolic states of dairy cows, which are closely related to their performance [35]. Just as lactose and solid nonfat in milk had significant different patterns among Sanhe dairy cattle with parities of 1–4, several serum biochemical indices were also found to have apparent differences that distinguished primiparous and multiparous Sanhe dairy cattle in this study. HDL, as a specific carrier of lipids, was present at higher levels in the S1 serum samples, but the serum HDL levels were within the normal range for all parities [36]. It should be noted that further studies are needed to determine whether significantly higher blood ammonia accumulation in the S1 group affects the performance of primiparous Sanhe dairy cattle in the presence of consistent dietary conditions, as no difference in the blood urea nitrogen content was observed among the four parities.

### 4.2. Association and Transformation of Amino Acids, Carbohydrates, and Lipids between Serum and Milk Samples from Sanhe Dairy Cattle with Parities of 1–4

In this study, the serum and milk samples from Sanhe dairy cattle with parities of 1–4 were comprehensively tested using untargeted metabolomics analyses. We found that these two types of bodily fluids’ metabolic status significantly differed between primiparous and multiparous Sanhe dairy cattle. A total of 36 shared metabolites were identified from the serum and milk of Sanhe dairy cattle with parities of 1–4. These metabolites were involved in the three nutritional categories of carbohydrates, lipids, and proteins, closely related to the synthesis of nutrients in milk.

Protein is one of the most valuable nutrients in milk [37]. As important components of protein, the content and proportion of amino acids directly affect the quality of milk. The analysis of amino acids in hydrolyzed milk showed differences between parities for individual amino acids, such as methionine, tyrosine, and proline. Although these amino acids were all at higher levels in the S1 group, the TAA level and EAA/TAA ratio were consistent with the trend observed for the milk protein levels, with the group of primiparous cows (group S1) showing lower levels than the multiparous cows represented by groups S2 and S3. In addition to proline, other amino acids had the highest content in the S1-B group. The fact that proline was found at the highest level in the milk samples in the S1 group, but at the lowest level in the serum samples in the S1 group confirms that there is a synergistic relationship between nutrient conversion in the blood and milk, as mentioned above [38]. However, unlike the notable differences observed in the essential amino acid (EAA) levels and the EAA/TAA ratio in milk between primiparous and multiparous cows, we did not observe significant changes in the EAA levels in the serum based on parity. This discrepancy may be attributed to the utilization of EAAs by the mammary gland for the synthesis of milk protein, which is concurrently influenced by blood glucose and insulin levels [39]. Our findings align with the positive correlation identified between the serum glucose levels and milk protein content. Additionally, key metabolites such as ketoleucine, L-kynurenine, and N-Acetylornithine, which strongly correlate with non-essential amino acids (NEAAs) in serum, participate in the amino acid metabolic pathway. This pathway represents one of the contributing factors influencing the variance in amino acid synthesis from the blood to milk protein among Sanhe dairy cattle with parities ranging from 1 to 4. Furthermore, the lower total protein levels detected in the serum for the S1 group compared to the S2, S3, and S4 groups were consistent with the results observed for the milk protein levels, further emphasizing a robust correlation between the blood nutrient metabolism and raw milk synthesis. Overall, our results suggest a linkage between the quality of raw milk and the metabolic state of nutrients in the blood, influenced by parity. However, additional samples and data analyses are essential to reliably determine whether the EAA content contributes to the observed differences in the milk protein content between primiparous and multiparous Sanhe dairy cattle. Previous studies have reported that feeds with higher EAA proportions improve protein utilization, thus being considered more nutritious [40,41]. Furthermore, tryptophan and cysteine, the two EAAs with the smallest proportions in milk [42], are easily destroyed under acidic conditions; therefore, the levels of these two amino acids cannot be determined using hydrolyzed milk samples. However, in our metabolome analysis results, tryptophan and its intermediate metabolites, including L-kynurenine, L-formylkynurenine, and N-acetylserotonin, were detected at higher levels in the S1-M group. There are studies that have reported that lactating cows afflicted with mastitis exhibit lower concentrations of tryptophan and kynurenine in their milk compared to healthy cows, suggesting these compounds could serve as potential biomarkers for mastitis [43]. Therefore, combined with the results of the present study, these findings suggest that more attention should be paid to the prevention and detection of mastitis in primiparous dairy cows.

Lactation is a physiological activity with a great energy demand [44]. As a direct source of energy for cattle, glucose is an essential precursor for lactose synthesis in lactating mammary glands [45]. Approximately 60–85% of the glucose transported to the mammary gland re-enters the bloodstream [46], while 65–70% of the glucose absorbed by the mammary gland is utilized for lactose synthesis [47]. Compared with multiparous Sanhe dairy cattle, primiparous Sanhe dairy cattle had lower serum glucose levels, despite having higher lactose levels. Previous studies have found that the relationship between the blood glucose levels and milk lactose levels is biphasic, which may be a reason for the differences in milk quality between cows of different parities [45], especially the difference in lactose levels. Although the glucose content in the blood significantly impacts lactose synthesis, glucose is not the only precursor of lactose synthesis, as galactose is an alternative carbon source for lactose synthesis [45]. This study showed a strong correlation between D-glucose and D-galactose in milk, and the serum galactose levels were highest in the S1 group, which may also be one reason for the differences in lactose levels among the different parities.

In addition to proteins and sugars, lipids are important nutrients in various metabolic activities. We did not identify significant differences in the fat content of the raw milk samples in Sanhe dairy cattle of different parities. However, the metabolomics results reflected differences in lipid metabolism between primiparous and multiparous Sanhe dairy cattle. Consistent with the lower serum triglyceride levels in primiparous cows than in multiparous cows, the levels of glycerol 3-phosphate, a key precursor required for triglyceride synthesis in mammalian cells [48], were also lower in the milk samples from cows in the S1 group. Higher levels of linoleic acid and its oxidative metabolite, 9(S)-HPODE [49], and lower levels of myristic acid and palmitic acid were detected in the milk from cows in the S1 group. In addition, the serum cholesterol levels were significantly higher in the S1 group, which is consistent with the trend of the metabolome results of cholesterol. Sphinganine, which is also involved in lipid metabolism [50], showed higher levels in the S1-B group. However, sphinganine and cholesterol did not show a synergistic trend in the milk samples from cows of different parities, which may be due to other pathways in the metabolic process that make the proportion of mammary gland utilization inconsistent [51]. Although the above differences in lipid metabolism between primiparous and multiparous Sanhe dairy cattle did not affect the total milk fat content, they still provided insights into the synthesis and composition of specific lipids in the milk of Sanhe dairy cattle.

## 5. Conclusions

This study elucidates the intricate relationship between the metabolic profile and production performance of Sanhe dairy cattle across different parities. Our findings underscore significant variations in milk traits and metabolites in both the milk and serum, highlighting the influence of parity on dairy cow metabolism. Specifically, multiparous cows exhibited distinct metabolic patterns compared to primiparous ones, with notable differences in milk composition and serum biochemical parameters. The identification of differential metabolites associated with key metabolic pathways provides valuable insights into the underlying mechanisms influenced by dietary supplementation and parity. Furthermore, the discovery of potential marker metabolites offers promising avenues for distinguishing between cows of different parities. To enhance the production performance of primiparous Sanhe cattle, it is recommended to strengthen the supply of energy and essential amino acids, ensuring optimal milk quality. This study underscores the importance of tailored nutritional strategies based on parity-specific metabolic profiles to optimize milk production and quality in Sanhe dairy cattle, thereby promoting sustainable dairy farming practices.

## Figures and Tables

**Table 1:** Body weight, dry matter intake, lactation performance, and hydrolyzed amino acid content of Sanhe dairy cattle with parities of 1–4.

Item ^1^	Group ^2^				SEM ^3^	*p*-Value
	S1	S2	S3	S4		
BW (kg)	569	572	591	575	10.26	0.889
DMI (kg/d)	21.88	21.28	21.79	21.86	0.30	0.430
Milk yield (kg/d)	29.43	30.56	31.68	30.85	2.64	0.937
Somatic cell count (×1000 cells/mL)	29.10	33.78	31.40	40.10	8.51	0.793
Milk fat content (%)	4.30	4.06	4.05	4.38	0.32	0.828
Milk protein content (%)	3.14 ^b^	3.49 ^a^	3.52 ^a^	3.13 ^b^	0.09	0.001
Lactose content (%)	5.24 ^a^	4.93 ^b^	4.99 ^b^	4.97 ^b^	0.07	0.009
Solid not fat (%)	9.41 ^a^	9.31 ^a^	9.28 ^a^	8.97 ^b^	0.09	0.011
Urea nitrogen content (%)	18.99	17.38	18.53	19.22	0.78	0.458
Total solids (%)	11.71	11.56	11.50	10.98	0.35	0.342
Free amino acid content (g/100 mL)						
EAA	1.41	1.62	1.64	1.39	0.074	0.076
Arginine	0.10	0.09	0.10	0.08	0.006	0.135
Threonine	0.13	0.14	0.13	0.11	0.006	0.273
Valine	0.19	0.24	0.23	0.20	0.011	0.203
Methionine	0.07 ^a^	<0.01 ^b^	0.05 ^ab^	0.04 ^ab^	0.009	0.009
Isoleucine	0.17	0.20	0.20	0.17	0.008	0.182
Leucine	0.29	0.35	0.34	0.29	0.015	0.166
Phenylalanine	0.15	0.18	0.18	0.15	0.010	0.087
Lysine	0.23	0.33	0.30	0.26	0.016	0.093
Histidine	0.09	0.10	0.10	0.08	0.004	0.101
NEAA	1.65	1.59	1.61	1.35	0.091	0.077
Aspartate	0.22	0.25	0.25	0.21	0.012	0.144
Serine	0.14	0.15	0.13	0.11	0.009	0.428
Glutamate	0.51	0.65	0.63	0.53	0.037	0.265
Glycine	0.06	0.06	0.06	0.06	0.003	0.088
Alanine	0.10	0.12	0.11	0.10	0.005	0.126
Tyrosine	0.09 ^a^	0.03 ^b^	0.08 ^a^	0.06 ^ab^	0.013	0.003
Proline	0.54 ^a^	0.34 ^b^	0.34 ^b^	0.29 ^b^	0.044	0.001
TAA	3.06	3.21	3.25	2.74	0.158	0.085
EAA/TAA	0.46 ^b^	0.51 ^a^	0.51 ^a^	0.51 ^a^	0.008	0.019

^1^ Abbreviations: BW, body weight; DMI, dry matter intake; EAA, essential amino acids; NEAA, non-essential amino acids; TAA, total amino acid; EAA/TAA, the ratio of essential amino acids to total amino acids. ^2^ S1, S2, S3, and S4 represented first-, second-, third-, and fourth-parity Sanhe dairy cattle, respectively. ^3^ SEM was standard error of means. ^a,b^ Means within a row with different superscripts differ significantly (*p* < 0.05).

**Table 2:** Serum biochemical parameters and free amino acid content of Sanhe dairy cattle with parities of 1–4.

Item ^1^	Group ^2^				SEM ^3^	*p*-Value
	S1	S2	S3	S4		
Total protein (g/L)	76.94 ^b^	83.24 ^a^	84.45 ^a^	81.15 ^ab^	1.79	0.017
Albumin (g/L)	42.11	42.87	41.56	40.24	1.06	0.337
Glucose (mM)	3.61 ^b^	3.88 ^a^	3.88 ^a^	3.84 ^a^	0.07	0.017
Blood urea nitrogen (mM)	6.54	6.01	6.15	6.74	0.38	0.477
Triglyceride (mM)	0.13 ^b^	0.16 ^a^	0.16 ^a^	0.13 ^b^	0.01	0.010
Cholesterol (mM)	5.95	5.71	5.77	5.02	0.31	0.154
Low-density lipoprotein (mM)	3.00	2.96	2.96	2.49	0.35	0.399
High-density lipoprotein (mM)	4.38 ^a^	4.26 ^a^	4.16 ^ab^	3.58 ^b^	0.22	0.047
Serum ammonia (μM)	319.17 ^a^	250.62 ^b^	268.43 ^b^	242.08 ^b^	13.30	0.002
Lipase (U/L)	17.85	19.31	19.75	19.59	0.98	0.608
Free amino acid content (μg/mL)						
EAA	151.74	157.67	148.90	143.14	0.15	0.324
Threonine	34.82	32.49	30.78	30.33	2.46	0.534
Valine	26.31	28.71	27.77	25.54	0.43	0.343
Methionine	5.13 ^a^	4.72 ^ab^	4.45 ^ab^	3.45 ^b^	1.22	0.024
Isoleucine	13.13	14.06	12.55	12.38	1.01	0.180
Leucine	16.40	18.75	16.58	15.65	1.08	0.126
Phenylalanine	8.57	8.66	7.40	7.96	0.32	0.115
Lysine	14.44	15.28	14.81	14.43	1.23	0.844
Histidine	9.24	10.40	10.18	9.27	0.39	0.120
NEAA	106.75	99.94	107.15	93.76	0.60	0.053
Aspartate	2.20 ^a^	1.56 ^b^	1.93 ^ab^	1.74 ^ab^	0.90	0.034
Serine	9.27	9.08	9.33	8.86	0.53	0.887
Glutamate	26.17	24.03	25.66	23.40	0.38	0.482
Glycine	27.87 ^a^	24.04 ^ab^	25.94 ^b^	22.81 ^ab^	0.78	0.006
Alanine	23.05 ^a^	18.98 ^ab^	21.41 ^ab^	18.56 ^b^	0.42	0.034
Cysteine	2.95	3.35	3.27	2.07	0.95	0.081
Tyrosine	8.79	8.42	7.60	7.58	1.10	0.249
Arginine	23.71	24.59	24.37	24.13	8.22	0.872
Proline	6.46 ^b^	10.48 ^ab^	12.01 ^a^	8.74 ^ab^	5.66	0.014
TAA	258.50	257.61	256.05	236.90	3.64	0.372
EAA/TAA	0.59	0.61	0.58	0.60	0.01	0.057

^1^ Abbreviations: EAA, essential amino acids; NEAA, non-essential amino acids; TAA, total amino acid; EAA/TAA, the ratio of essential amino acids to total amino acids. ^2^ S1, S2, S3, and S4 represented first-, second-, third-, and fourth-parity Sanhe dairy cattle, respectively. ^3^ SEM was standard error of means. ^a,b^ Means within a row with different superscripts differ significantly (*p* < 0.05).

## Data Availability

The authors confirm that the data supporting the findings of this study are available within the article.

## Supplementary Materials

The following supporting information can be downloaded at: https://www.mdpi.com/article/10.3390/metabo14040227/s1, Supplementary file 1.docx, Supplementary file 2.xlsx.
