# Supplementary material for: Combined Metabolomics and Biochemical Analyses of Serum and Milk Revealed Parity-Related Metabolic Differences in Sanhe Dairy Cattle
Source: Metabolites. 2024 Apr 16;14(4):227. doi: 10.3390/metabo14040227 (PMC11052102; doi:10.3390/metabo14040227)
Supplement: Supplementary file 1 [file metabolites-14-00227-s001.zip › Supplementary_file_1.pdf]

Supplementary file 1

Combined Metabolomics and Biochemical Analyses of Serum and Milk Revealed Parity-Related Metabolic Differences in Sanhe Dairy Cattle

Zixin Liu [1,2,†], Aoyu Jiang [1,2,†], Xiaokang Lv [1,2,3], Dingkun Fan [1], Qingqing Chen [1], Yicheng Wu [1,2], Chuanshe Zhou [1,2,*] and Zhiliang Tan [1,2]

[1] Key Laboratory for Agro-Ecological Processes in Subtropical Region, Institute of Subtropical Agriculture, Chinese Academy of Sciences, Changsha 410125, China; liuzixin20@mails.ucas.ac.cn (Z.L.); jiangaoyu21@mails.ucas.ac.cn (A.J.); lvxk@ahstu.edu.cn (X.L.); 82101221244@caas.cn (D.F.); 2018391005@st.gxu.edu.cn (Q.C.); wuyicheng19@mails.ucas.ac.cn (Y.W.); zltan@isa.ac.cn (Z.T.)

[2] University of the Chinese Academy of Sciences, Beijing 100049, China

[3] College of Animal Science, Anhui Science and Technology University, Bengbu 233100, China

* Correspondence: zcs@isa.ac.cn; Tel.: +86-731-84619795; Fax: +86-731-84612685

† These authors contributed equally to this work.

Table S1

Ingredients and chemical composition of experimental diets.

Item	Content
Ingredients, % of diet DM	
Soybean meal	13
Cottonseed meal	2
Barley	5
Distillers Dried Grains with Solubles	2
Sprouting corn bran	2
Corn	21
Flaked maize	5
Pelleted beet pulp	2
Cottonseed	5.5
Oat hay	2.5
Alfalfa hay	11
Corn silage	23
Alfalfa silage	4.5
NaHCO3	0.5
Premix1	1
Total	100
Nutrient levels[2]	
DM	48.85
CP	15.14
NDF	35.22
ADF	37.57
EE	6.83
Ash	10.23
GE (MJ/kg)	16.56
Ca	0.78
P	0.36

[1] The premix provided the following per kg of diets：50 g Mg, 2.5 g Fe, 0.4 g Cu, 2 g Mn, 1.5 g Zn, 10 mg Se, 25 mg I, 5 mg Co, 500,000 IU vitamin A, 25,000 IU vitamin D, and 2500 IU vitamin E.

[2] DM = dry matter; CP = crude protein; NDF = neutral detergent fiber; ADF = acid detergent fiber; EE= ether extract; GE = gross energy; Ca = calcium; P = phosphorus.

Table S2

Multivariate statistical analysis parameters from untargeted metabolomic of Sanhe dairy cattle with a parity from to 1−4 .

Item	Statistical model[1]	R2X(cumulative)[2]	R2Y(cumulative)[3]	Q2(cumulative)[4]
Serum	PCA	0.530		
	PLS-DA	0.658	0.983	0.919
Milk	PCA	0.522		
	PLS-DA	0.644	0.971	0.939

[1] PCA, principal component analysis; PLS-DA, partial least squares discriminant analysis.

[2] R2X means the rate of interpretation of the X matrix by the model.

[3] R2Y means the rate of interpretation of the Y matrix by the model.

[4] Q2 represents the predictive ability of the model.

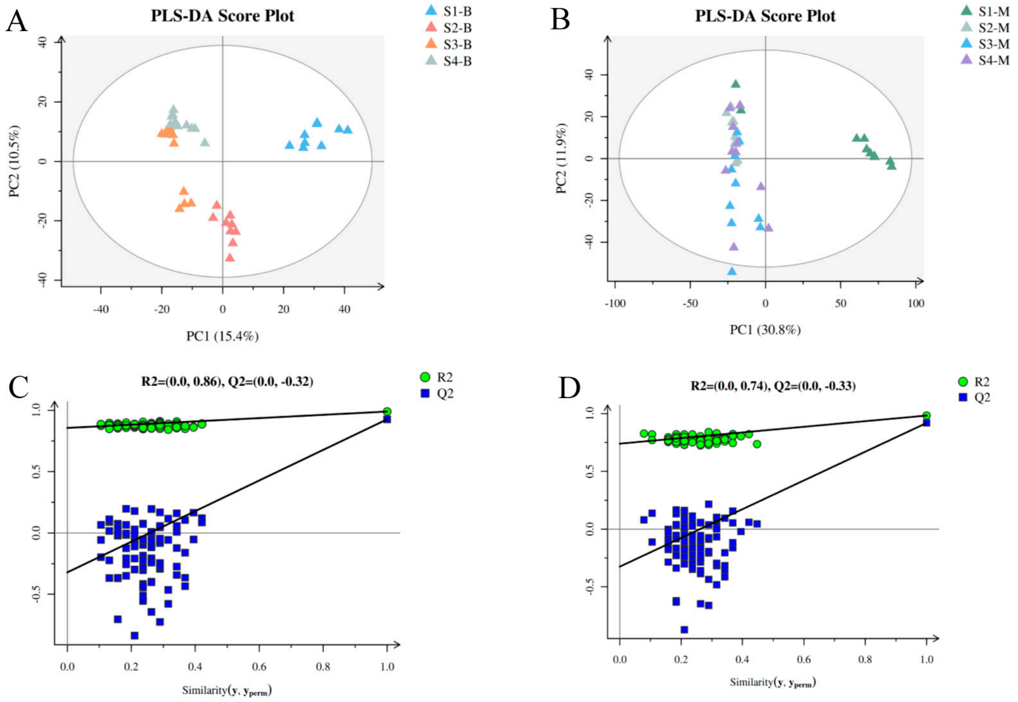

Fig. S1. (A) Principal component analysis (PCA) score plot of serum samples from S1 - S4 based on untargeted metabolomics. (B) PCA score plot of milk samples from S1 - S4 based on untargeted metabolomics. (C) Permutation test plots of serum samples from S1 - S4. (D) Permutation test plots of milk samples from S1 - S4. The abscissa PC1 = first principal component and the ordinate PC2 = second principal component. Q2 = percentage of Y dispersions predicted by the model using cross-validation; R2 = percentage of Y dispersions explained by the model.

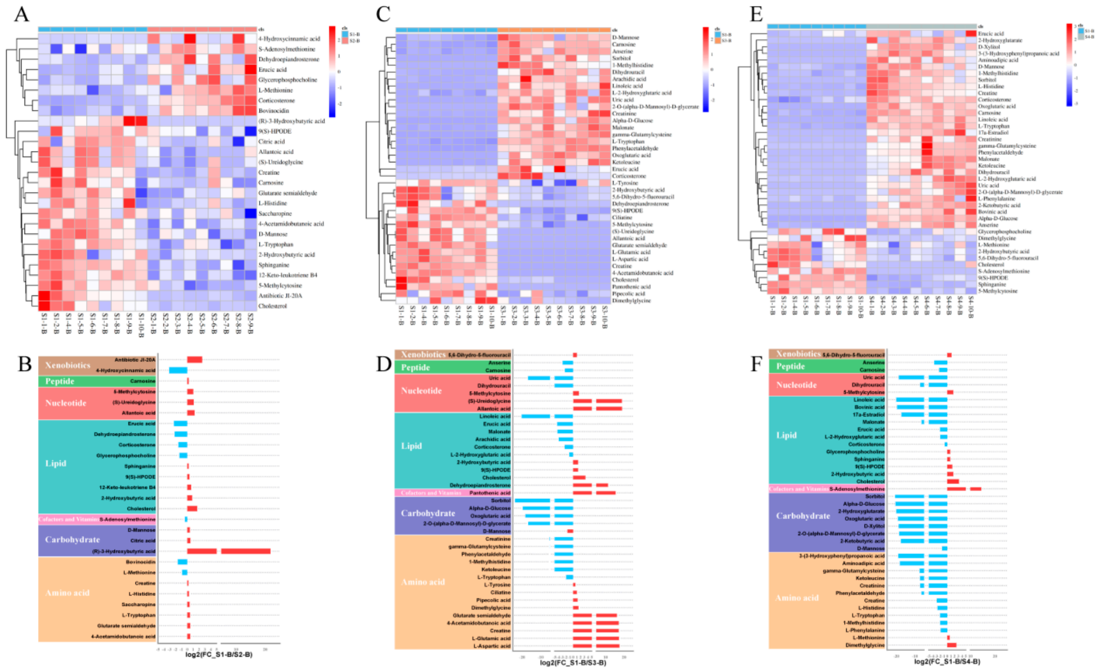

Fig. S2. (A), (C), and (E) HCA of differential metabolites identified from S1-B vs. S2-B, S1-B vs. S3-B, and S1-B vs. S4-B comparisons, respectively. (B), (D), and (F) Classification of differential metabolites from S1-B vs. S2-B, S1-B vs. S3-B, and S1-B vs. S4-B groups, respectively, based on KEGG pathway analysis and the analysis of fold-change values for each differential metabolite. Different colored blocks represent the corresponding metabolic category classification. Red bars indicate higher levels of the metabolites identified in the S1-B group and blue bars indicate lower levels of metabolites identified in the S1-B group.

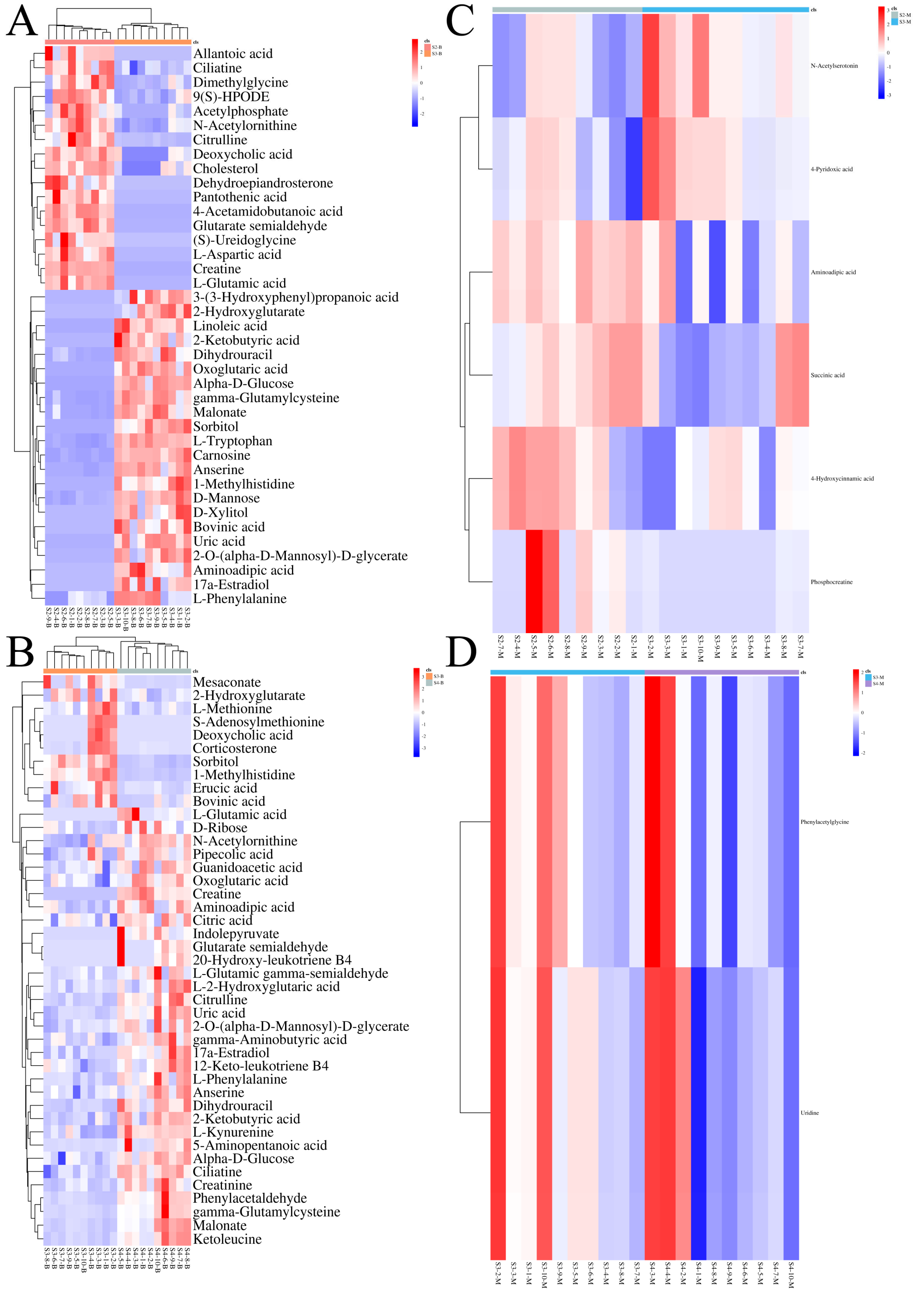

Fig. S3. (A) Hierarchical cluster analysis (HCA) of differential metabolites identified from S2-B vs. S3-B. (B) HCA of differential metabolites identified from S3-B vs. S4-B. (C) HCA of differential metabolites identified from S2-M vs. S3-M. (C) HCA of differential metabolites identified from S3-M vs. S4-M.

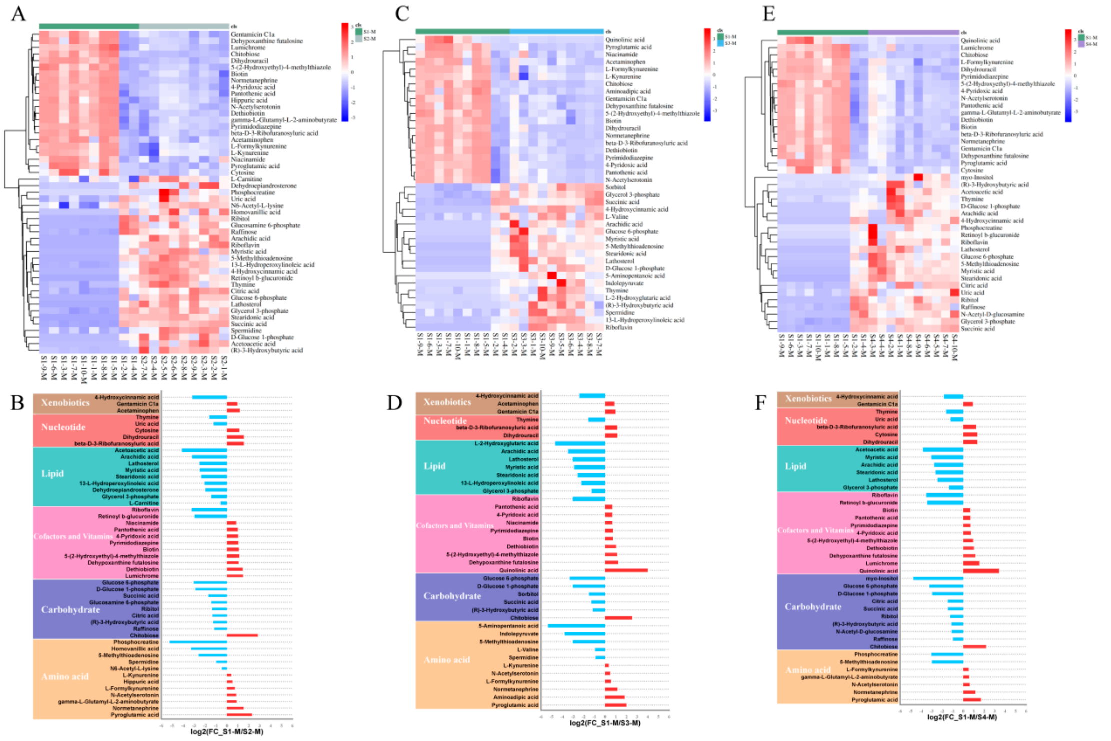

Fig. S4. (A), (C), and (E) HCA of differential metabolites identified from S1-M vs. S2-M, S1-M vs. S3-M, and S1-M vs. S4-M comparisons, respectively. (B), (D), and (F) Classification of differential metabolites from S1-M vs. S2-M, S1-M vs. S3-M, and S1-M vs. S4-M comparisons, respectively, based on KEGG pathway analysis and the analysis of fold-change values for each differential metabolite. Different colored blocks represent the corresponding metabolic category classification. Red bars indicate higher levels of metabolites identified in the S1-M group and blue bars indicate lower levels of metabolites identified in the S1-M group.

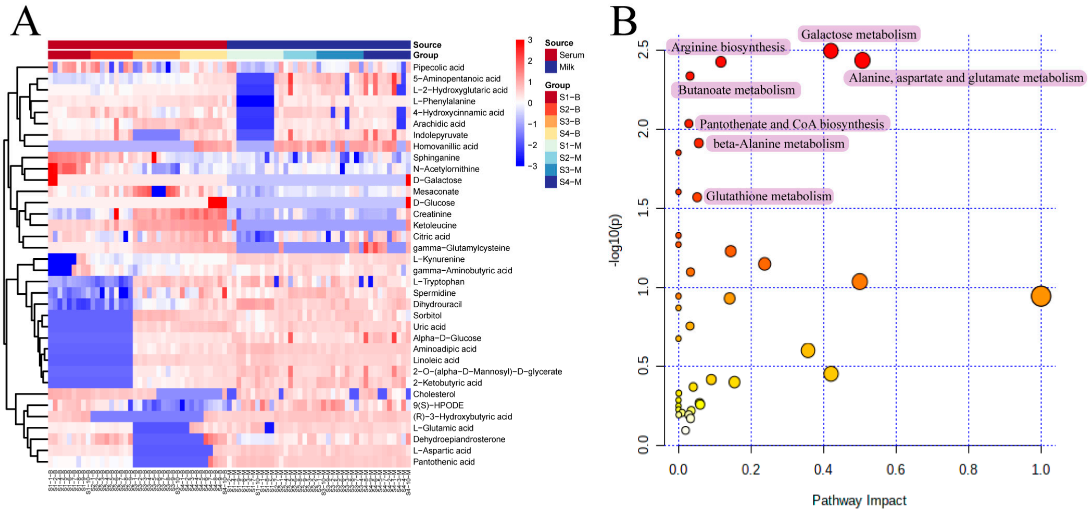

Fig. S5. (A) Hierarchical cluster analysis of differential metabolites co-identified in serum and milk samples. The red bars represent serum samples, and the blue bars represent milk samples. Red and blue bars represent serum-derived and milk-derived metabolites, respectively. (B) Bubble chart analysis of functional pathways from co-identified metabolites in serum and milk samples. x-axis, pathway impact; y-axis, -log (P). Circles represent metabolic pathways. Darker circles indicate more significant changes in the metabolite levels in the corresponding pathway, whereas the circle size corresponds to the pathway impact score. Significantly enriched functional pathways are marked with purple blocks.

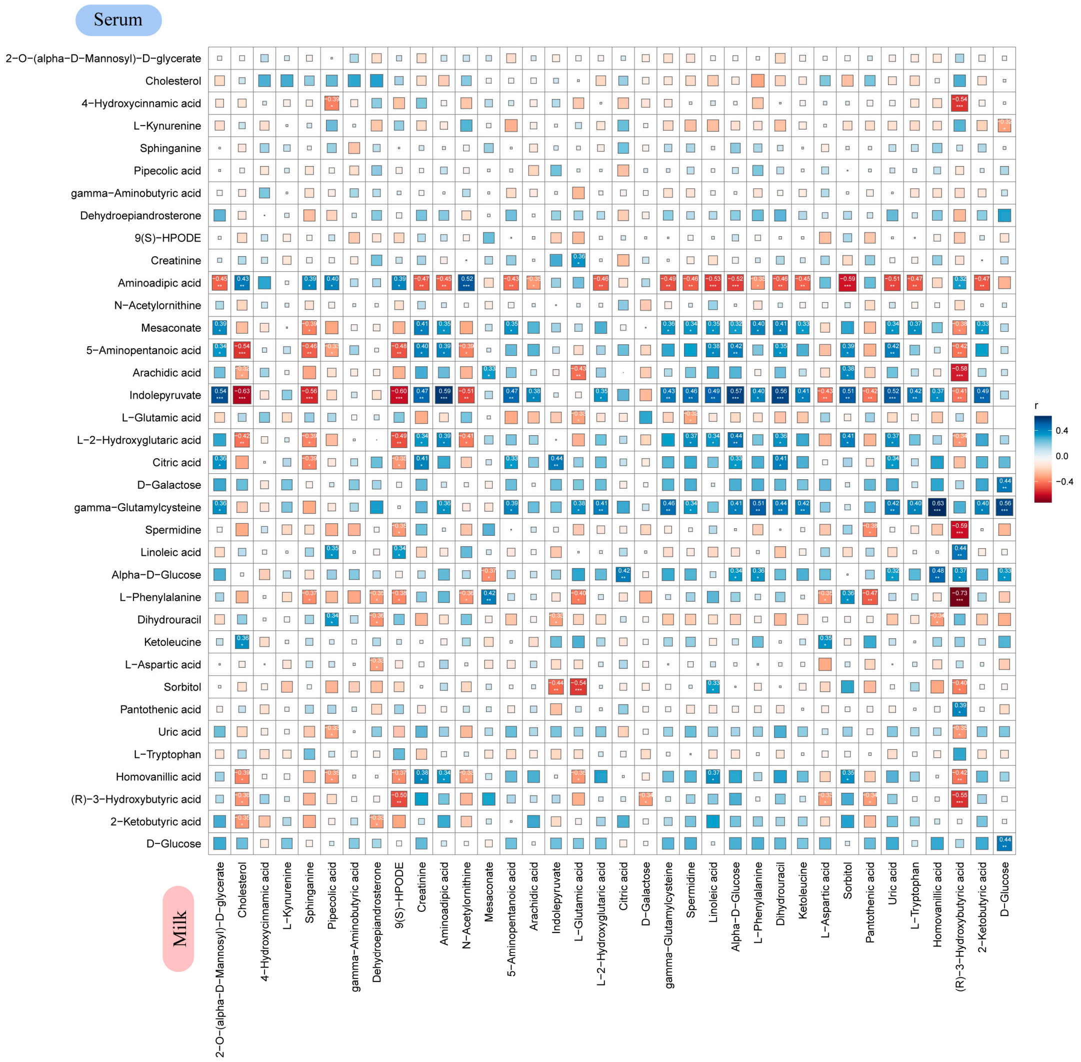

Fig. S6. Spearman's correlation analyses of shared differential metabolites in serum and milk. The x-axis represents the shared metabolites in milk from S1-S4 and the y-axis represents the shared metabolites in serum from S1-S4 . Box size and colour gradient indicate the strength of Spearman's correlation, with blue indicating a positive correlation and red a negative correlation; white characters in the boxes indicate the correlation coefficients, with * indicating 0.01 < P < 0.05, ** indicating 0.001 < P < 0.01, and *** indicating P < 0.001.

Analysis code

Functional pathway enrichment analysis

Functional pathway enrichment analysis was performed using the tools at Metaboanalyst 5.0 (http://www.metaboanalyst.ca/). Applicable to Fig.5A, Fig.5B, Fig.5C, Fig.5E, Fig.5F, Fig.5G. Take Fig. 5A as an example for the specific code (R version 4.0.2).

```
mSet<-InitDataObjects("conc", "pathora", FALSE)
cmpd.vec<-
c("KEGG","C00836","C02140","C17704","C05669","C02376","C08316","C00670",
"C00187","C00073","C00449","C01227","C00019","C14827","C00811","C05984","
C01089","C05949","C00078","C02946","C00499","C00159","C02091","C03273","C
00158","C00135","C00300","C00386")  #Similarly, the KEGG number of
corresponding differential metabolites in other pairwise comparison groups can be
input here
mSet<-Setup.MapData(mSet, cmpd.vec);
mSet<-CrossReferencing(mSet, "kegg");
mSet<-CreateMappingResultTable(mSet)
mSet<-SetKEGG.PathLib(mSet, "bta", "current")
mSet<-SetMetabolomeFilter(mSet, F);
mSet<-CalculateOraScore(mSet, "rbc", "hyperg")
mSet<-PlotPathSummary(mSet, T, "path_view_2_", "png", 72, width=NA, NA, NA )
```

```
mSet<-SaveTransformedData(mSet)
```

Hierarchical cluster analysis

Hierarchical cluster analysis of differential metabolites co-identified from serum and milk. Advanced Heatmap Plots was performed using the OmicStudio tools at https://www.omicstudio.cn. Upload csv format data of differential metabolites with identified intensities in the corresponding experimental individuals for Fig. 3B, Fig. 3C, Fig. 3E, Fig. 3G, Fig. 4B, Fig. 4C, Fig. 4E, Fig. 4G, Fig. S1, Fig. S2. Take Fig.6B as an example for the specific code (R version 3.6.3).

```
mSet<-InitDataObjects("conc", "pathora", FALSE)
cmpd.vec<-c("Arachidic acid","13-L-Hydroperoxylinoleic acid","Acetoacetic acid","Chitobiose","Retinoyl b-glucuronide","Thymine","4-Hydroxycinnamic acid","5-Methylthioadenosine","Spermidine","Myristic acid","Stearidonic acid","Homovanillic acid","Pyroglutamic acid","Lathosterol","Cytosine","L-Carnitine","N6-Acetyl-L-lysine","Gentamicin C1a","Biotin","N-Acetylserotonin","Dethiobiotin","4-Pyridoxic acid","L-Formylkynurenine","L-Kynurenine","Niacinamide","Pyrimidodiazepine","beta-D-3-Ribofuranosyluric acid","gamma-L-Glutamyl-L-2-aminobutyrate","Normetanephrine","5-(2-Hydroxyethyl)-4-methylthiazole","Pantothenic acid","Lumichrome","Dehypoxanthine futalosine","Dihydrouracil","Hippuric acid","Acetaminophen","(R)-3-Hydroxybutyric acid","Riboflavin","D-Glucose 1-phosphate","Citric acid","Glucose 6-
```

```
phosphate","Succinic acid","Dehydroepiandrosterone","Phosphocreatine","Glycerol
3-phosphate","Uric acid","Glucosamine 6-phosphate","Ribitol","Raffinose")
#Similarly, the names of corresponding differential metabolites in other pairwise
comparison groups can be input here
mSet<-Setup.MapData(mSet, cmpd.vec);
mSet<-CrossReferencing(mSet, "name");
mSet<-CreateMappingResultTable(mSet)
mSet<-SetKEGG.PathLib(mSet, "bta", "current")
mSet<-SetMetabolomeFilter(mSet, F);
mSet<-CalculateOraScore(mSet, "rbc", "hyperg")
mSet<-PlotPathSummary(mSet, F, "path_view_0_", "png", 72, width=NA, NA, NA )
mSet<-PlotPathSummary(mSet, T, "path_view_1_", "png", 72, width=NA, NA, NA )
mSet<-PlotKEGGPath(mSet, "Synthesis and degradation of ketone bodies",576, 480,
"png", NULL)
mSet<-RerenderMetPAGraph(mSet, "zoom1647762835106.png",576.0, 480.0, 100.0)
```
